# Enhanced Photoluminescence Property for Quantum Dot-Gold Nanoparticle Hybrid

**DOI:** 10.1186/s11671-015-1067-0

**Published:** 2015-10-15

**Authors:** Qianqian Huang, Jing Chen, Jian Zhao, Jiangyong Pan, Wei Lei, Zichen Zhang

**Affiliations:** School of Electronic Science and Engineering, Southeast University, Nanjing, 210096 China; State Key Laboratory of Precision Measurement Technology and Instruments, Department of Precision Instrument, Tsinghua University, Beijing, 100084 China

**Keywords:** Quantum dot, Metal–semiconductor system, LSPR

## Abstract

In this paper, we have synthesized ZnCdSeS quantum dots (QDs)-gold nanoparticle (Au NPs) hybrids in aqueous solution via bi-functional linker mercaptoacetic acid (MPA). The absorption peaks of ZnCdSeS QDs and Au are both located at 520 nm. It is investigated that PL intensity of QD-Au hybrid can be affected by the amounts of Au and pH value of hybrid solution. The located surface plasmon resonance (LSPR) effect of QD-Au NPs has been demonstrated by increased fluorescence intensity. The phenomenon of fluorescence enhancement can be maximized under the optimized pH value of 8.5. LSPR-enhanced photoluminescence property of QD-Au hybrid will be beneficial for the potential applications in the area of biological imaging and detection.

## Background

Photoluminescence (PL) properties of CdSe quantum dots (QDs) have been extensively investigated due to its potential application such as solar cell [1, 2], light emitting devices [3], biological imaging [4] and so on. With the improvement in material nature, trying composite structure is another way to promote QDs’ properties. Recently, metal–semiconductor system has become research focus because of the complex interplay of enhancing and quenching physicochemical processes due to the metal surface plasmons (SPs). Both the theoretical and experimental study have been done for the promising applications in nanotechnology and biotechnology [5–11]. To enhance the luminescence, the metal SPs-enhanced fluorescence has been one of the most effective methods [5]. In the isolated metallic nanostructures, due to the collective oscillations of free electrons, the localized surface plasmon resonance (LSPR) is excited accompanied with the enhancement of optical near field [6]. Due to the existence of local electromagnetic field, the distance between the metal and QDs is demonstrated to be an influential factor to the fluorescence enhancement. For example, by using a layer-by-layer polyelectrolyte deposition technique, a distance-dependent enhancement and quenching of CdSe/ZnS core/shell QDs coupling with gold colloids has been observed [7]. The enhancement and quenching of single-molecule fluorescence has also been studied. In the process of gold particle closing to the fluorescent molecule, it is observed that the phenomenon of fluorescence has been enhanced first and then weakened with varied distance between the two particles [8]. Moreover, the coupling of a single molecule to a single spherical gold nanoparticle has been acted as a nanoantenna, and the evidence for the role of LSPR in the excitation and emission processes has been presented [9]. Due to the resonance energy transfer (RET) between donor and acceptor, researchers control the format of the metal NPs similar to the absorption of the acceptor to achieve greatly enhanced luminescence [10]. Additionally, not only the gold NPs, the metal nanoshell can also be used for molecular fluorescence experiments with better adjustability [11].

However, exploration of the molecular fluorescence enhancement is still so limited, especially in aqueous solution. Up to now, most studies on optical properties of metal–semiconductor system are based on solid state [12–15]. It is because highly control of the uniformity and dispersibility of the nanoparticles is required in aqueous solution. Different from the solid state, the QDs-metal hybrid presents special properties that can be available for nanotechnology and biotechnology in water phase. On the other hand, the PL-quenching phenomenon indicates the existence of other process in the metal–semiconductor system. Li et al. [12] investigated carrier dynamics of luminescence quenching in CdSe/ZnS core/shell QDs and emphasized the quenching phenomenon mainly caused by the fluorescence resonance energy transfer. And Yin et al. [13] proposed that one reason of the PL quenching in metal–semiconductor system is the reverse charge transfer from semiconductor to the metal at the inference. Therefore, to get the strongest enhancement and the weakest quenching is the most important thing to improve the PL properties of the metal–semiconductor system.

In this work, we shall present a novel technique for the synthesis of ZnCdSeS QDs-Au NPs hybrid in aqueous solution. LSPR enhancement for QD-Au hybrid can also be demonstrated.

## Methods

### Synthesis of OA-capped ZnCdSeS QDs

We firstly synthesized oleic acid (OA)-capped ZnCdSeS QDs in chloroform solution [3]. As a typical synthetic procedure, 0.2 mmol of CdO, 4 mmol of zinc acetate, 15.5 mmol of oleic acid (OA), and 30 mL of 1-octadecene were placed in a 100-mL round flask. The mixture was heated to 150 mol in the flowing high-purity N_2_ for 30 min and further heated to 300 °C to form a clear solution of Cd(OA)_2_ and Zn(OA)_2_. At this temperature, a stock solution containing 5 ml of trioctylphosphine, 0.2 mmol of Se, and 4 mmol of S was quickly injected into the reaction flask. After the injection, the reaction temperature was kept for 3 min for promoting the growth of QDs and then cooled down to room temperature to stop the growth. QDs were washed with acetone for three times and finally dispersed in chloroform at the concentration of 10 mg/ml.

### Preparation of MPA-capped ZnCdSeS QDs

MPA was chosen as a bi-functional linker to synthesize MPA-capped ZnCdSeS QDs [1]. A 7.5-mL purified OA-capped ZnCdSeS QDs in chloroform solution containing approximately 75 mg of ZnCdSeS QDs in powder form was firstly mixed with 15 mL mercaptoacetic acid (MPA) and 75 mL chloroform in a flask, which was stirred continuously for about an hour. After centrifugation at 6500 rpm for 5 min and the supernatant fluid being discarded, the resulting precipitate containing MPA-capped ZnCdSeS QDs was dissolved in distilled (DI) water and some tetramethylammonium hydroxide (TMAOH) was added to make it clear. The solution was then centrifuged again in the same way. The QDs are stable in the solution which the pH values range from 8 to 12. We controlled the concentrations of the QDs of 10 mg/ml.

### Preparation of Au NPs

The water soluble Au NPs were synthesized through the reduction of gold chloride with sodium citrate in aqueous solution [16]. Solutions were prepared of HAuCl_4_ (0.01 % by weight in DI water, solution A) and of Na_3_-citrate (0.5 % by weight in DI water, solution B). One hundred millilitre of solution A was heated to boiling, and 4 mL of solution B was then added. The colour of the boiling solution changed from blue to a brilliant red, which indicated the formation of spherical particles. After the boiling state maintaining for about 5 min, the reduction of gold chloride had almost completed.

### Preparation of QD-capped Au NPs Hybrid

The QD-Au hybrid was fabricated using the above QDs and Au NPs solution [12]. The QDs solution was firstly diluted to one ninth of the original concentration by using DI water and then taken 1 mL to add certain quantities of Au NPs. The quantity of Au NPs was varied from 0 to 120 μL. After about 5 h standing, the sample solution was completed. The QDs solution and Au NPs solution with the same concentration were prepared to compare with the sample solution. Here, we kept the quantities of QDs constant and only changed the volume of Au NPs solution.

### Sample and Device Characterization

The absorption and photoluminescence (PL) spectra were measured by U-4100 UV-visible and NIR-300 spectrophotometer, respectively. The TEM was equipped with a multiscan charge-coupled device (CCD) camera system (Model 894, Gatan, USA) to record the HRTEM images.

## Results and Discussion

**Fig. 1:**
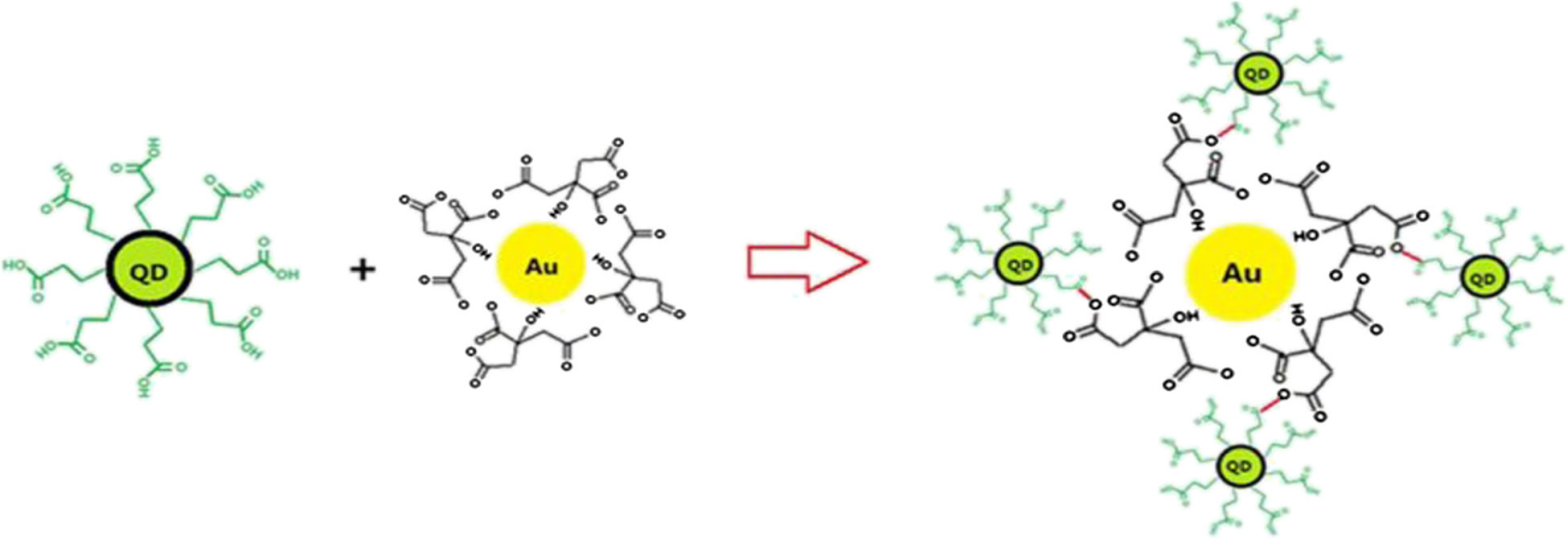
Construction process of QD-Au discrete nanostructures

Figure 1 shows the construction process of QD-Au discrete nanostructures, which vividly explains the progress of the formation of QD-Au hybrid. Firstly, OA-capped ZnCdSeS QDs in chloroform solution were synthesized. And then, by using MPA as the bi-functional linker, we synthesized MPA-capped ZnCdSeS QDs. Due to the functional groups with charging, QDs were positively charged in the water solution. On the other hand, the water soluble Au NPs were synthesized through the reduction of gold chloride with sodium citrate in aqueous solution. The Au NPs were negatively charged because of the citrate ions absorbing on the surface of the Au NPs. Naturally, the QDs and Au NPs connected together immediately as the two kinds of solutions were mixed [12].

**Fig. 2:**
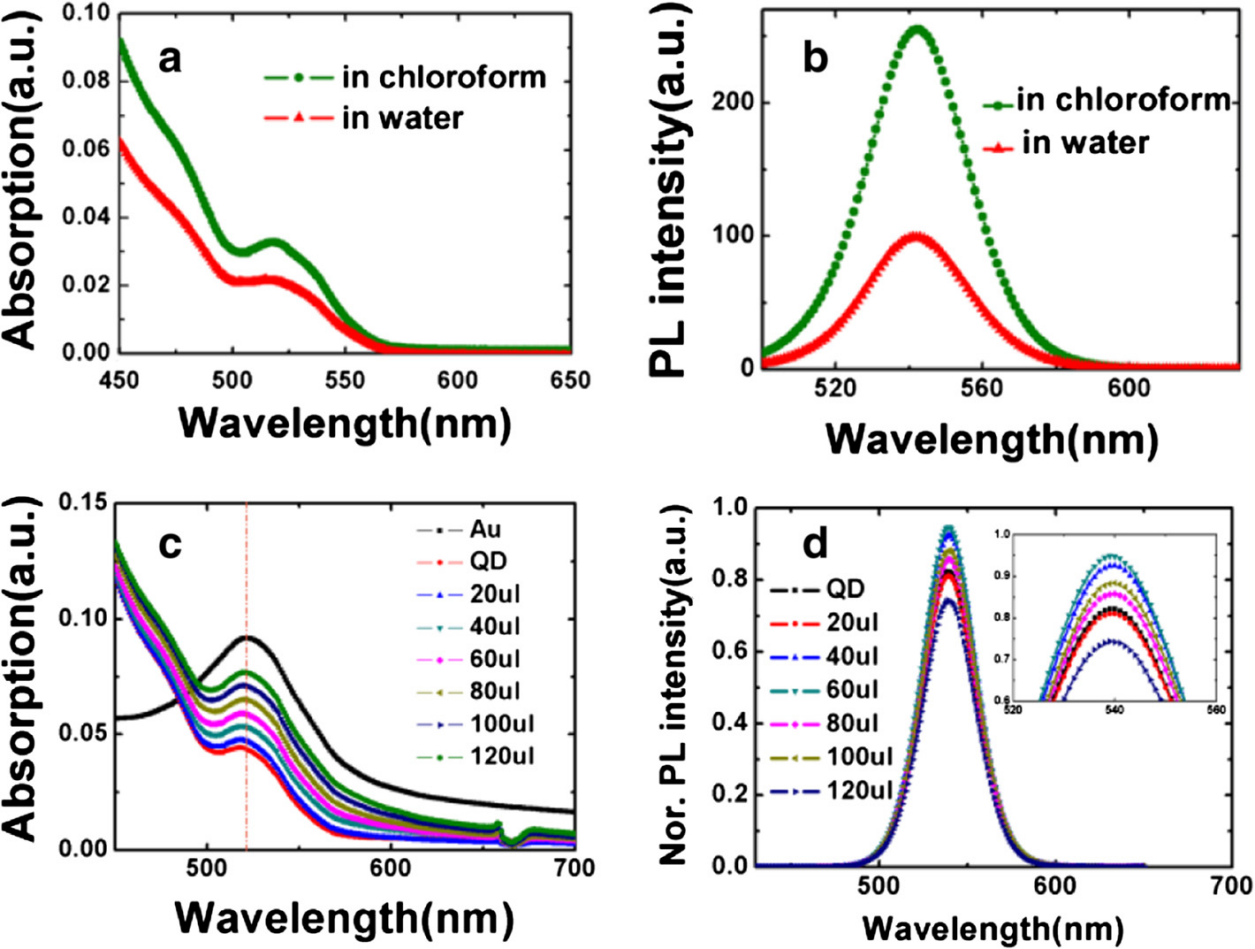
(Color online) **a** and **b** The absorption and PL intensity spectra of QDs in chloroform and in water (PH value = 8.5). **c** The absorption spectra of Au NPs individually and QDs-Au NPs conjugates. **d** PL intensity spectra of QDs-Au NPs hybrids. The quantity of Au NPs was varied from 0 to 120 μL both in (**c**) and (**d**)

Figure 2a, b presents the absorption spectra and the PL intensity spectra of OA-capped ZnCdSeS QDs in chloroform solution and MPA-capped ZnCdSeS QDs in aqueous solution (pH value = 8.5). The absorption spectra clearly display the first excitonic transition peak at 520 nm for the QDs in chloroform and aqueous solutions. It is indicated that QD size has not changed as the QD transfer from hydrophobic solution to hydrophilic solution. The PL emission was excited at 480 nm using a Xe lamp. The peaks were both located at 540 nm; however, the peak intensity of the latter sample became weaker than the former one. According to the characteristics of absorption and PL intensity spectra, it is found that the QDs size has not affected by different functional groups and solvents.

To investigate the amount of Au NPs on the LSPR effect, we prepared the ZnCdSeS QDs-Au hybrid with the quantity of Au NPs varied from 0 to 120 μL. Figure 2c shows the absorption spectra of bare Au NPs and QDs-Au hybrids. The spectra peak of Au NPs and QDs-Au NPs were both located at 520 nm. It is worth noting that the spectra peak of Au NPs is consistent to ZnCdSeS QDs (Fig. 2a), and the absorption peak intensity is increased with unchanged peak position as the amount of Au NPs increased.

The PL spectra of ZnCdSeS QDs with and without Au NPs excited at 365 nm are shown in Fig. 2d. The emission wavelengths were located at 540 nm regardless of the content of Au NPs. Additionally, the phenomenon of fluorescence enhancement and quenching was clearly observed. The peak value increased until 60 μL of Au NPs solution was added into QDs and then declined. The phenomenon described above indicates that the PL intensity of ZnCdSeS QD-Au hybrid is mainly determined by two processes [4, 12, 13]: (1) PL quenching due to the energy and the charge transfer between Au and the QDs or other nonradiative processes and (2) PL enhancement due to the enhancement of absorption and radiative rate induced by LSPR. The PL intensity of the QDs/Au composite system is the competing result of the two processes above. Obviously, as the amount of Au NPs reached to 60 μL, the second process was playing a major role, as a result of PL enhancement; as the amount of Au NP exceeded 60 μL, the first process dominated, leading to the PL quenching phenomenon.

**Fig. 3:**
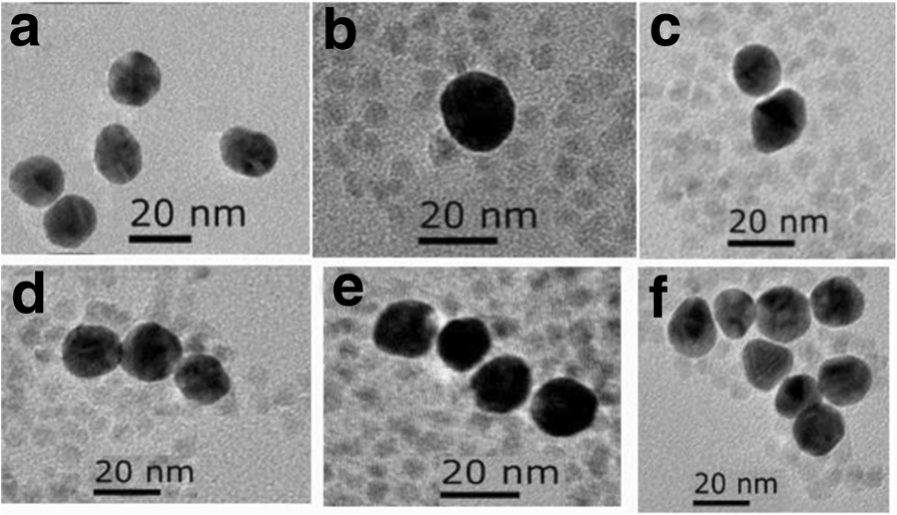
TEM images of **a** Au NPs, **b**–**f** QDs-Au NPs hybrids

Transmission electron microscopy (TEM) measurements were performed to obtain the size distribution by dropping each solution on carbon-coated copper grids. From Fig. 3a, it is presented that the average size of the Au NPs was about 20 nm. Figure 3b–f shows the different nanostructures of ZnCdSeS QDs-Au hybrids. It can be seen that Au NPs (dark black spots) are surrounded by QDs (light black spots) after mixing. The size of QDs is estimated as 5 nm. Besides of the situation of single Au NP surrounded by QDs, it occurs that a number of Au NPs were reunited together and were then wrapped by QDs. It is observed that when the amount of Au NPs of 20 μL was added into QDs, it is likely to form single Au surrounded by QDs, shown as Fig. 3b. As the amount was Au NPs of 40 μL, double Au NPs surrounded by QD are occurred in Fig. 3c. As the amount of Au NPs was 60 μL, the circumstances of three Au NPs surrounded by QDs were happened in Fig. 3d. As the amount of Au NP increases to 80 μL, four Au NPs surrounded by QDs were in the arrangement as Fig. 3e. As the higher amount of Au NPs was mixed with QDs, more Au NPs as the cluster surrounded by QDs were shown as Fig. 3f. According to Fig. 2d, phenomenon of fluorescence enhancement and quenching for QD-Au hybrids is originated from the formed nanostructure of Au NPs and QDs.

**Fig. 4:**
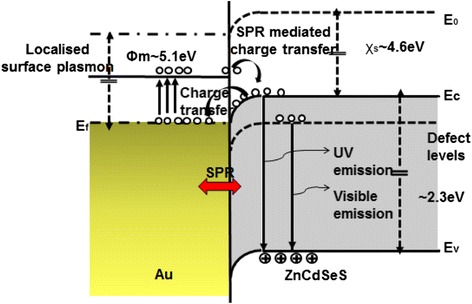
Model representing the mechanism behind NBE enhancement in ZnCdSeS quantum dots (QDs)-gold nanoparticle (Au NPs) hybrids

Figure 4 shows the Model representing the mechanism behind near-band edge emission (NBE) enhancement in ZnCdSeS quantum dots (QDs)-gold nanoparticle (Au NPs) hybrids. In the QDs, the separated carriers would recombine in two ways: one is the direct recombination which contributes to the surface exciton emission and another one is that the excess holes at the surface tunnel into the deep level centers inside the QDS and produce defect-related emission. The surface exciton emission is relatively weak due to the small electron–hole wave function overlap, making the defect bound exciton emission from the bulk region of QDs dominate the luminescence spectra. When the QDs are directly coated with gold NPs, since the work function for gold is lower than that for QDs, electrons will migrate from Au to the conduction band of QDs to achieve the Fermi level equilibrations, which results in an increased surface exciton emission. On the other hand, as the energy level for the donor-like defects, when excited the direct contact between the metal and semiconductor will facilitate the transfer of electrons from the metal NPs to the defects in QDs, enhancing the defect-related emission. The two processes would lead to the PL quenching [13, 17–19].

**Fig. 5:**
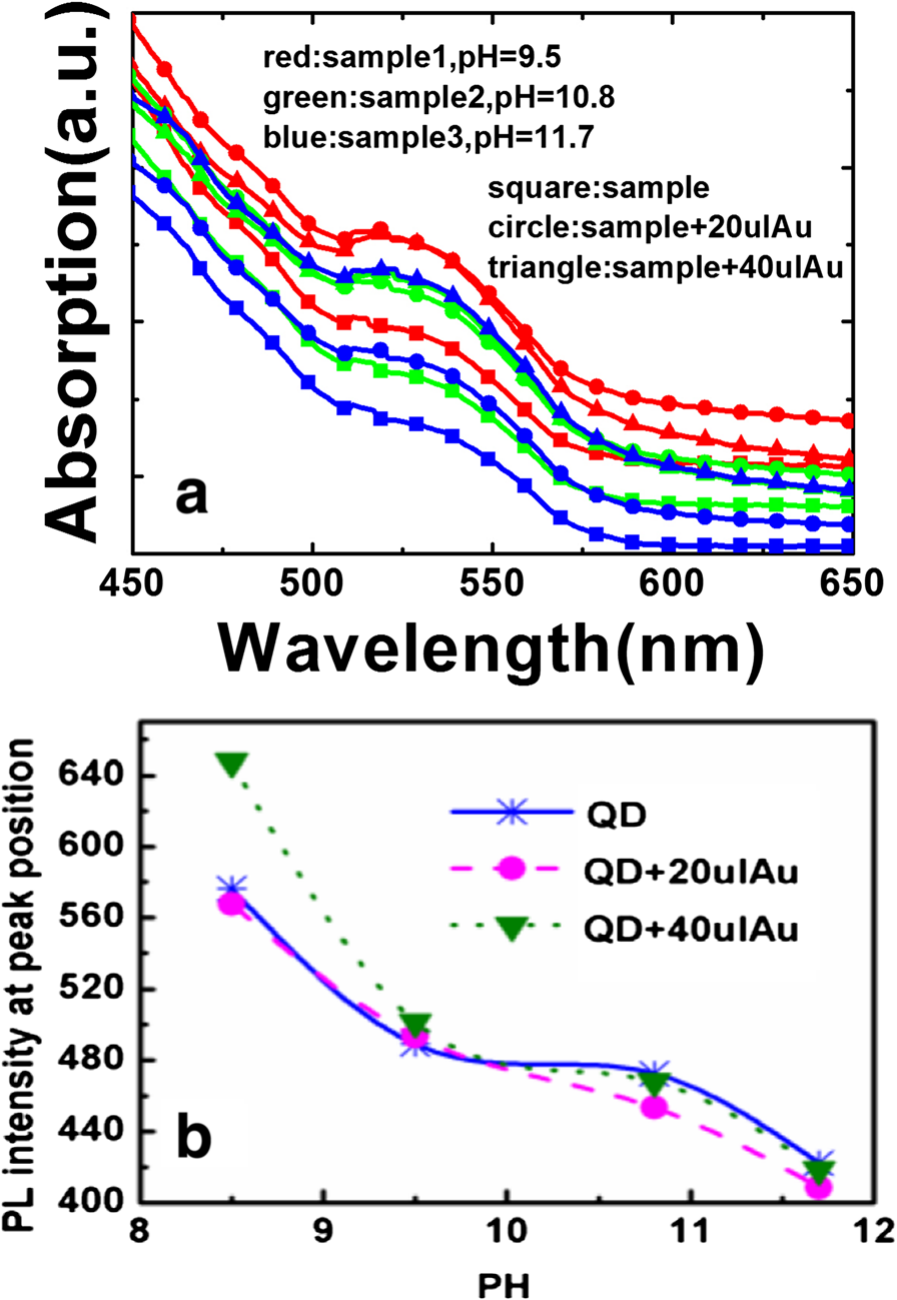
**a** The absorption spectra **b** peak fluorescence intensity of QD-Au hybrid in aqueous solution (pH value =8.5, 9.5, 10.8, 11.7)

To study the influence of pH value to the PL enhancement of hybrids, we carried out a series of experiments (pH value = 9.5, 10.8, 11.7). The absorption spectra (Fig. 5a) clearly display the first excitonic transition peak at about 520 nm for the QDs in different solutions, which indicates that the pH value of the solution cannot affect the excitonic transition peak for the QDs. Figure 5b shows the PL intensity spectra of MPA-capped ZnCdSeS core/shell QDs in aqueous solutions (pH value = 9.5, 10.8, 11.7). The PL emission was excited at 365 nm using a Xe lamp. It is found that, as the pH value increased, the PL value was decreased. The absorption spectra remain nearly unchanged (Fig. 5a), providing evidence that no particle growth occurs. At the mean time, the emission peak has not been shifted after adding of Au solution.

Figure 5b shows peak fluorescence intensity of QD-Au hybrid solutions in aqueous solution (pH value = 8.5, 9.5, 10.8, 11.7). The fluorescence intensity of the hybrid can be enhanced by decreasing the pH value of the solution. The highest PL value occurs under pH value of 8.5. It can be deduced that a secondary coordination at the CdSe particle surface exists, which in return provides better surface passivation and consequently higher PL efficiency [17]. After adding Au solution, the enhancement phenomenon can also be observed, suggesting an interaction between the QDs and Au NPs exists due to the LSPR from Au NPs. However, the enhancement phenomenon cannot be observed anymore with the increased pH value. It can be explained by that the combination between Au and QDs is affected due to the changing pH value, which contains the LSPR from Au NPs to QDs. Under the condition of pH value of 8.5, the highest PL intensity can be achieved. Meanwhile, it is observed that the emission peak has red-shifted, which was attributed to an additional reaction taking place on the QD surface during the increase of the pH value [20, 21].

## Conclusions

Highly efficient ZnCdSeS QDs-Au hybrids have been synthesized via connecting with bi-functional linker. It is demonstrated that Au NPs can result in PL enhancement of ZnCdSeS QDs due to metal LSPR, which depends on the amount of Au NPs and pH value of solution. As the amount of Au NPs increased, the PL intensity reached the maximum value, then decreased, which was reverent with the morphology of QD-Au hybrid. In addition, PL intensity was also affected by pH value of the hybrids. The phenomenon of PL enhancement can be maximized under the pH value of 8.5.
